# Plasmonic efficiencies of nanoparticles made of metal nitrides (TiN, ZrN) compared with gold

**DOI:** 10.1038/srep38647

**Published:** 2016-12-09

**Authors:** Adrien Lalisse, Gilles Tessier, Jérome Plain, Guillaume Baffou

**Affiliations:** 1 LNIO/ICD, UMR 6281, CNRS, Technological University of Troyes, 10004, Troyes, France; 2 Laboratoire de Neurophotonique UMR8250, CNRS, Faculté des sciences biomédicales et fondamentales, Université Paris Descartes, 75270, Paris, France; 3 Institut Fresnel, CNRS, Aix Marseille Univ, Centrale Marseille, Marseille, France

## Abstract

Metal nitrides have been proposed to replace noble metals in plasmonics for some specific applications. In particular, while titanium nitride (TiN) and zirconium nitride (ZrN) possess localized plasmon resonances very similar to gold in magnitude and wavelength, they benefit from a much higher sustainability to temperature. For this reason, they are foreseen as ideal candidates for applications in nanoplasmonics that require high material temperature under operation, such as heat assisted magnetic recording (HAMR) or thermophotovoltaics. This article presents a detailed investigation of the plasmonic properties of TiN and ZrN nanoparticles in comparison with gold nanoparticles, as a function of the nanoparticle morphology. As a main result, metal nitrides are shown to be poor near-field enhancers compared to gold, no matter the nanoparticle morphology and wavelength. The best efficiencies of metal nitrides as compared to gold in term of near-field enhancement are obtained for small and spherical nanoparticles, and they do not exceed 60%. Nanoparticle enlargements or asymmetries are detrimental. These results mitigate the utility of metal nitrides for high-temperature applications such as HAMR, despite their high temperature sustainability. Nevertheless, at resonance, metal nitrides behave as efficient nanosources of heat and could be relevant for applications in thermoplasmonics, where heat generation is not detrimental but desired.

New plasmonic materials are attracting a strong interest due to recent advances in nanoplasmonics related to high-temperature applications^1^,^2^, light harvesting^3^,^4^, thermoplasmonics^5^,^6^,^7^,^8^,^9^, heterogeneous catalysis^4^,^10^, sensing^11^ and active plasmonics^12^,^13^. This new trend reduces the supremacy of gold and silver in plasmonics^14^,^15^,^16^,^17^,^18^,^19^,^20^,^21^.

Metal nitrides (TiN, ZrN and HfN) are among the most promising new materials in plasmonics^6^,^22^,^23^,^24^. They offer three main benefits: (i) they feature plasmonic properties similar to gold, (ii) their plasmonic properties are tunable *via* the metal/nitrogen stoichiometry that can be varied and last but not least (iii) they are *refractory* materials^25^. This adjective means that they can sustain high temperatures without melting. For instance, the melting point of TiN is 2930 °C and ZrN 2952 °C, while the melting point of gold is only 1064 °C, at atmospheric pressure. This important feature opens the path for applications where the temperature of the plasmonic device is likely to be high under operating conditions, such as in heat assisted magnetic recording.

Magnetic recording (MR), or magnetic storage, consists in storing binary information on a ferromagnetic film^26^. This concept, at the basis of common hard disk drives, is currently limited to an areal density of 1 Tbit · in^−2^ 27. This limitation mainly comes from the instability of small magnetic grains undergoing thermal agitation. The smaller the grain volume, the higher the probability of spontaneous demagnetization (even below the Curie temperature) and loss of information. Logic suggests that shrinking the grain volume could be compensated by increasing the coercivity of the material. However, too high a coercivity value would require non-realistic magnetic field amplitudes to act on the magnetic dipole and write data, or would require prohibitive energy consumption. A promising approach to circumvent this limitation consists in benefiting from the temperature dependence of coercivity, which is a decreasing function. Local heating of the magnetic disk upon writing is thus expected to enable data storage on high-coercivity materials, and therefore higher densities. This strategy has been investigated since the 1980s and is called heat-assisted magnetic recording (HAMR)^1^,^2^. It is, however, not commercialized yet. Ideally, the heat deposition has to be confined over a spatial scale smaller than 50 nm and made without contact between the disk and writing head. These requirements suggest the relevance of an optical method to heat the magnetic substrate by light absorption over a sub-diffraction-limit area.

In 2006, Matsumoto and coworkers, from the Hitachi company, proposed for the first time the use of plasmonic nanostructures as optical near-field enhancers in HAMR^28^,^29^,^30^. Their idea was to benefit from the ability of metal nanotips to create a strong and confined optical field in their vicinity, which can be used to very locally heat the substrate by a photothermal transduction over an area below the diffraction limit. Thus, using such a near-field transducer (NFT), *it is not the temperature increase within the metal nanostructure itself that is involved in the heating mechanism, but rather the optical near-field*. Today, plasmonic-assisted HAMR is becoming an active area of research, as a novel application in plasmonics^26^,^31^,^32^,^33^.

One of the major problems in HAMR remains the heat generation within the metal NFT attached to the writing head under the intense laser illumination required in HAMR. Gold has been the material of choice for NFTs so far^31^. However, gold nanoparticles are known to reshape upon heating, even at temperatures lower than the melting point of gold. Reshaping is observed typically between 100 °C and 400 °C^34^,^35^,^36^. The lifetime of NFTs may suffer from this problem. For this reason, efforts are made to find new plasmonic materials that can sustain higher temperatures, such as metal nitrides.

Titanium nitride (TiN) has been the most investigated metal nitride in plasmonics so far. Introduced in 2011 by the group of Boltasseva^22^, it is foreseen to be a good candidate to constitute the metal NFT of the writing head in HAMR applications. Today, the plasmonic capabilities of metal nitrides as compared to gold are not well established. On the one hand, the figure of merit −*ε*′*/ε*″ is often utilized to simply predict the plasmonic efficiencies of plasmonic materials^16^,^19^,^37^. But this metrics only yields a rough estimation and does not permit quantitative comparison between several materials^38^. On the other hand, some quantitative comparisons have been reported between Au and TiN, even experimentally^39^. However, it only concerned specific morphologies (discs). Finally, a large variety of plasmonic materials, including Au, TiN and ZrN have been recently compared theoretically using novel figures of merit^38^. However, the latter work only focused on metal nanoparticles in the quasistatic regime, *i.e.* on small nanoparticles where retardation effects are not involved. The quasistatic approximation fails in the case of arbitrary large structures, and numerical simulations become mandatory.

In this article, numerical simulations related to Au, TiN and ZrN nanoparticles are conducted with the aim of comparing the efficiency of these materials for applications in nano-optics. For this purpose, an important parameter is the nanoparticle morphology. To address this aspect of the problem, this article focuses on spheroid nanoparticles of various sizes and aspect ratios, in order to consider the effects of retardation (*i.e.* size) and asymmetries. The near-field enhancement and the heat generation were calculated as functions of the morphology. A last part of the article is dedicated to discussing the possible fields of applications for metal nitrides according to our results, with a particular focus on HAMR.

## Results

### Numerical model

In order to comprehensively compare the localized plasmon properties of different materials, many parameters have to be varied: the shape, the size, the surrounding refractive index and the incoming light wavelength. The shape is the most complicated parameter to investigate. On the one hand, one cannot reasonably investigate all the possible nanoparticle morphologies. One the other hand, trying to derive some general rules by sticking to dipolar spheres would be misleading. Nanoparticle asymmetries and retardation effects are very likely to play an important role.

A sensible approach to investigate the effect of the morphology is to consider spheroids of various sizes and aspect ratios. This way, the number of morphological free parameters is limited to only two and the main features acting on a plasmonic resonance are addressed: (i) a symmetry breaking from a spherical shape (such as with rods and discs) and (ii) an increase of the size of the nanoparticle (to induce retardation effects). We opted for this approach in this work as detailed below.

**Figure 1:**
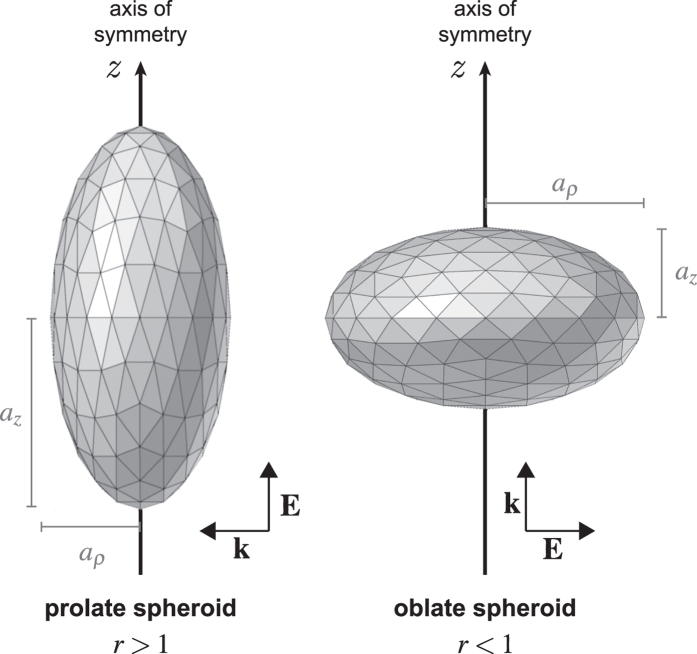
Meshes of the spheroids used for the simulations using the Boundary Element Method^40^. They were composed of 3840 vertices and 1280 faces. Depending on the morphology (prolate or oblate), the polarization of the incident light was different in order to match the longer dimension of the spheroid in any case.

The morphology of the nanoparticle considered in this article is described in Fig. 1. The meshes used for the simulation using the Boundary Element Method (BEM)^40^ are represented. Optical constants for gold and titanium nitrides were taken respectively from refs 41 and 42. Matlab codes are provided in Supplementary Materials. Prolate (*i.e.* cigar-like) and oblate (*i.e.* pumpkin-like) spheroids have been investigated. In both cases, the axis of symmetry is the *z* axis. A spheroid is defined by two parameters *a*_*ρ*_ and *a*_*z*_ where *a*_*ρ*_ is the spheroid semi-axis length along the *x* and *y* axes and *a*_*z*_ the semi-axis length along the *z* axis. The equation of the spheroid in cartesian coordinates reads


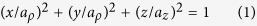


The aspect ratio of the spheroid is defined by


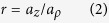


with this convention, a prolate spheroid corresponds to *r* > 1 and an oblate spheroid corresponds to *r* < 1. Finally, the equivalent radius, defined by the radius of a sphere of equal volume, is


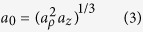


A spheroid can be defined either by the set of parameters *a*_*ρ*_ and *a*_*z*_, or by the set of parameters *a*_0_ and *r*. In the following, we will rather use the parameters *a*_0_ and *r* to characterize a spheroid.

For spheroid nanoparticles, the polarization of the incoming light naturally matters. In our numerical simulations, depending on the nature of the spheroid (prolate or oblate), the orientation of the incoming electric field will be different, so that it always excites the nanoparticle along its longest dimension. As represented in Fig. 1, for a prolate spheroid (*r* > 1), using complex notations, the incoming electric field reads


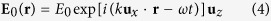


and for an oblate spheroid (*r* < 1)


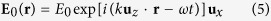


*k* = 2*πn*/*λ* where *n* is the refractive index of the surrounding medium. In all our simulations, we considered *n* = 1.33. This is the refractive index of water, a common surrounding medium, but this also stands for an average refractive index of materials in optics. Let us note **E**(**r**) the complex amplitude of the total electric field at any location of space **r**. We define the near-field enhancement factor Fa by


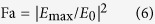


where *E*_max_ is the maximum value of |**E**(**r**)| achieved over the space outside the nanoparticle volume. The way it is defined, Fa corresponds to the Faraday parameter defined in ref. 38.

Along with Fa, the second parameter of interest that will be systematically calculated in our simulations is the temperature increase Δ*T* of the nanoparticle. Calculating Δ*T* requires the knowledge of the delivered power *P* = *σ*_abs_*I*, which requires itself the determination of the absorption cross section *σ*_abs_. *σ*_abs_ can be determined by standard optical simulation means used in nanoplasmonics, for instance the Boundary Element Method (BEM). However, calculating Δ*T* from *P* is a thermodynamic problem. Whereas it is straightforward for spherical nanoparticles^43^ (Δ*T* = *P*/4*πκa*), it is not that simple for a nanoparticle of arbitrary morphology. Interestingly, the thermodynamic problem of calculating Δ*T* has formal analogy with an electrostatic problem^44^. Temperature and electrostatic potential are both governed by the Poisson equation, which becomes the Laplace equation in the absence of sources: ∇^2^*A* = 0. The temperature, the thermal conductivity and the heat source density in thermodynamics become the electric potential, the permittivity and the charge density in electrostatics. In particular, the problem of an electric conductor of charge *Q* is equivalent to the problem of an object of infinite thermal conductivity, heated by a power *P*. In electrostatics, one has *U* = *Q*/*C*_el_ where U is the electrostatic potential of the object and *C*_el_ its electric capacitance. In thermodynamics, the equivalent relation reads^44^


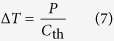


where *C*_th_ is similar to a thermal capacitance. For instance, for a sphere of radius *a*, *C*_el_ = 4*πε*_0_*a* in electrostatics, while in thermodynamics:


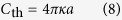


Determining the electric capacitances of *spheroids* is a problem that has been solved a long time ago in terms of formal integral expressions^45^. Fortunately, close-form expressions for spheroids have been derived more recently^46^ and they can be directly used to determine the *thermal* capacitances needed in this study. The expressions are different depending on the nature of the spheroid (prolate or oblate). Let us define the Laplace radius *a*_L_ such that the thermal capacitance for any morphology can be still written using the sphere-like expression^43^:


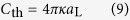


For prolate spheroids (*r* > 1), the Laplace radius reads


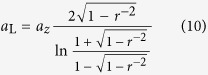


For oblate spheroids (*r* < 1), the Laplace radius reads


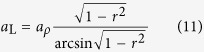


**Figure 2:**
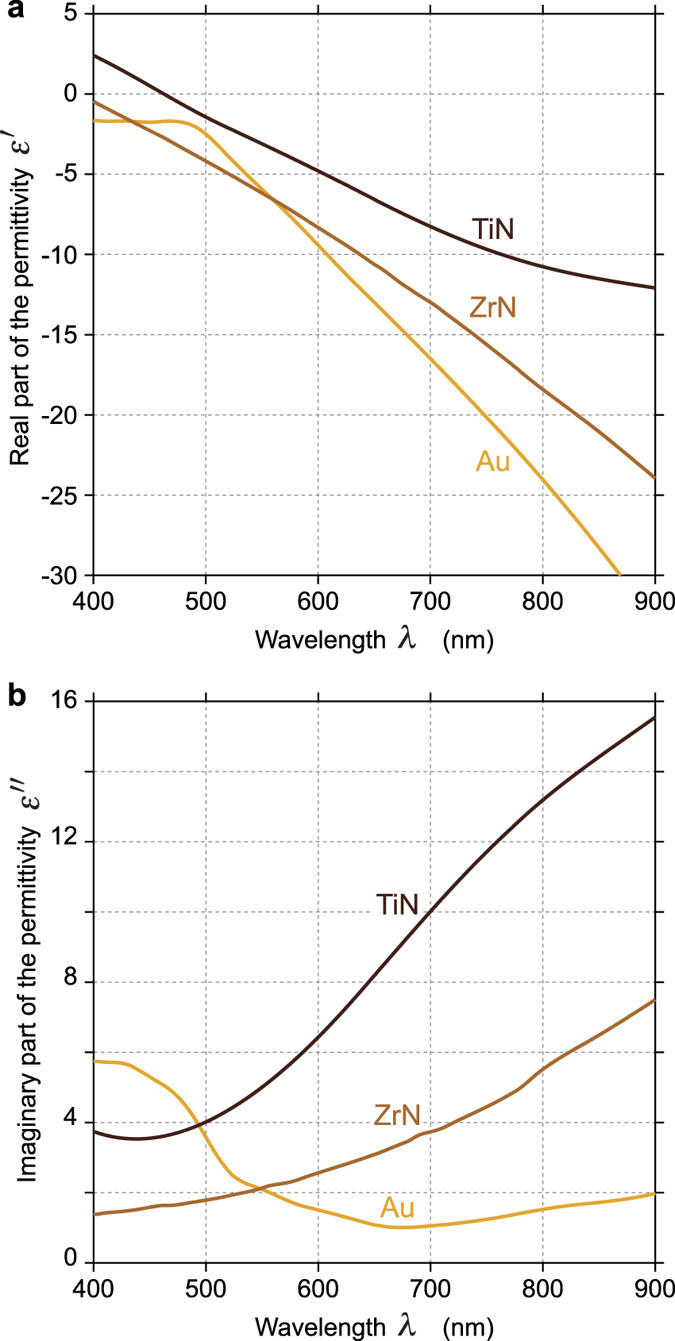
Real (**a**) and imaginary (**b**) parts of the permittivity of Au, TiN and ZrN as a function of the wavelength calculated from data sets of Johnson and Christy^41^ (for gold) and Palik’s Handbook^42^ (for metal nitrides).

In all the numerical simulations presented in this article, the complex permittivity of Au was taken from Johnson and Christy^41^ (known to be much more reliable than Palik’s values), and the permittivities of TiN and ZrN were taken from Palik’s handbook^42^. The real and imaginary parts of these permittivities are plotted as a function of the wavelength in Fig. 2.

### Calculations of nanoparticle efficiencies

**Figure 3:**
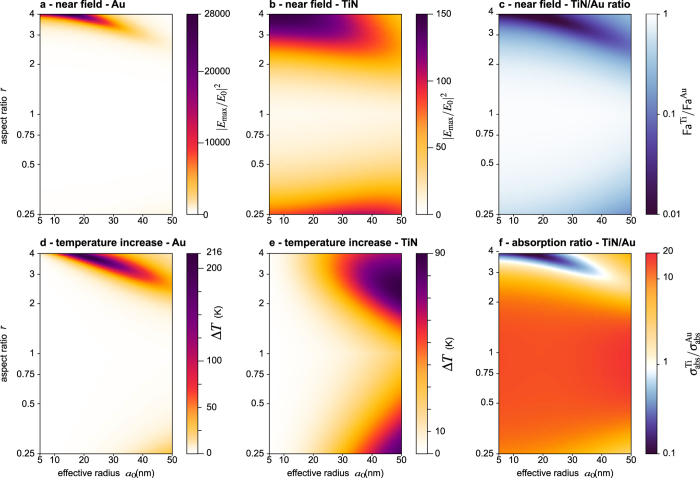
Properties of Au and TiN spheroids as a function of the aspect ratio *r* and the equivalent radius *a*_0_, under illumination at *λ* = 800 nm. (**a**) Near-field enhancement of Au spheroids. (**b**) Near-field enhancement of TiN spheroids. (**c**) Ratio of the data represented in images (**a**,**b**) respectively. (**d**) Temperature increase of Au spheroids. (**e**) Temperature increase of TiN spheroids. (**f**) Ratio of the data represented in images (**d**,**e**), which amounts to representing the ratio of the absorption cross sections of TiN and Au spheroids.

**Figure 4:**
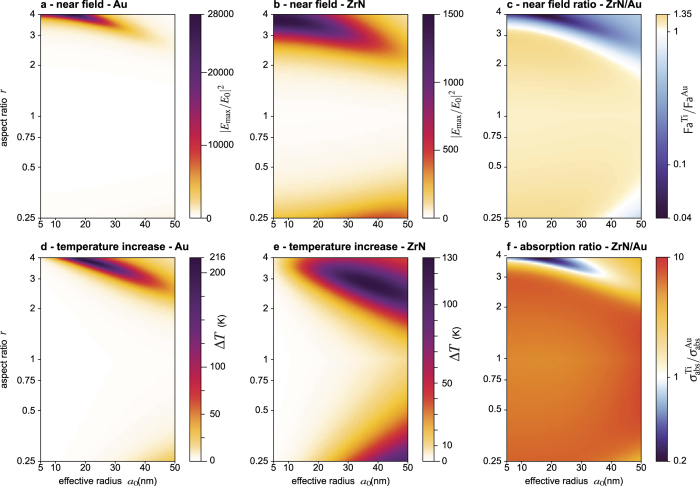
Properties of Au and ZrN spheroids as a function of the aspect ratio *r* and the equivalent radius *a*_0_, under illumination at *λ* = 800 nm. (**a**) Near-field enhancement of Au spheroids (reproduced from Fig. 3a). (**b**) Near-field enhancement of TiN spheroids. (**c**) Ratio of the data represented in images (**a,b**). (**d**) Temperature increase of Au spheroids (reproduced from Fig. 3d). (**e**) Temperature increase of ZrN spheroids. (**f**) Ratio of the data represented in images (**e**,**d**), which amounts to representing the ratio of the absorption cross sections of ZrN and Au spheroids.

The values of Δ*T* (for an arbitrary reference light intensity of 1 mW · m^−2^) and Fa fully characterize the plasmonic efficiencies of a nanoparticle for most applications. We have calculated these values for a large range of nanoparticle sizes *a*_0_, aspect ratios *r* and wavelengths *λ*. Representing results involving three free parameters is difficult. For this reason, we chose to vary *a*_0_ and *r*, but we fixed the wavelength because varying the morphology and sticking to a given wavelength matches common experimental investigations. We conducted numerical simulations at *λ* = 500, 650, 800 nm. Although all the results are discussed within this article, only the results at 800 nm are plotted herein (Fig. 3 for TiN and Fig. 4 for ZrN), because it is a common wavelength used experimentally, in particular for HAMR^28^,^29^,^30^,^31^,^32^,^33^. Results at 500 nm and 650 nm, discussed hereinafter, are gathered in Supplementary Figs S1–S4.

The first line of Fig. 3 (Fig. 3a–c) concerns the near-field enhancement characterized by the Faraday number Fa, for TiN and Au nanoparticles. Figure 3a,b show that the resonances in near-field enhancement of Au and TiN occur over the same (*a*_0_, *r*) space of values. This observation confirms and illustrates the similarities commonly put forward between gold and titanium nitride regarding their plasmonic properties. The resonances differ, however, in amplitude and width. While the near-field enhancement around gold nanoparticles can be gigantic, especially for prolate shapes (like rods), the near-field enhancement of TiN remains very weak in comparison. This is clearly observed in Fig. 3c, which displays the ratio of the near-field enhancements between TiN and Au (*i.e.* the ratios between the maps of Fig. 3a,b respectively). Interestingly, even out of resonance, TiN never does better than gold. TiN only equals Au performances for spherical shapes, far from the resonance, when the near-field enhancements are minimal, which is a shame. This is expected, because any metal sphere features the same near-field enhancement of Fa = 9 at sufficiently large wavelength (corresponding to the quasi-static regime)^38^.

The second line of Fig. 3 (Fig. 3d–f) concerns the temperature increase Δ*T* experienced by the TiN and Au spheroids. For gold, the magnitude of Δ*T* follows the near-field enhancement. It is strong around an aspect ratio *r* of 3 to 4, and weakens upon enlarging the nanoparticle. For TiN, the variations of Δ*T* are different. On the contrary, for large nanoparticles (large values of the effective radius *a*_0_), Δ*T* is larger. The ratio of Δ*T* for TiN and Au is represented in Fig. 3f. It amounts to representing the ratio between the absorption cross sections *σ*_abs_ of the nanoparticles. Interestingly, although TiN nanoparticles were not able to outperform Au nanoparticles regarding the near-field enhancement, they stand for very efficient heat sources compared to gold. For a given morphology, the temperature of a TiN nanoparticle is always larger than the temperature of a gold nanoparticle, except at the resonance of the gold nanoparticle (blue area on Fig. 3f). This gigantic and narrow resonance of gold compared to metal nitrides, especially in the near-infrared, is due to the weak value of its imaginary part, as evidenced in Fig. 2b. Noteworthily, although heat generation and loss in plasmonics are related to the imaginary part of the permittivity, a reduced imaginary part of the permittivity leads to a larger heat generation. This counterintuitive relation is explained in ref. 38.

Following these results, it is worth discussing a recent experimental work addressing the photothermal efficiency of TiN compared to gold. In 2013, Guler and coworkers studied TiN nanoparticles as nanosources of heat, both experimentally and numerically^39^. They showed that TiN nanodiscs can demonstrate higher heat generation compared to gold nanodiscs at 800 nm. This observation is consistent with our results, and in particular with Fig. 3f: when considering oblate particles, like discs, TiN always outperforms gold. This is however not the case of prolate particles. Consequently, the observation and the conclusion reported by Guler and coworkers may be related to the choice of discs (*i.e.* oblate) morphologies. According to our results, if the authors had conducted their studies on rod-like (*i.e.* prolate) shapes, it is likely that their conclusion would have been the opposite: poorer capabilities of TiN compared to gold.

The same numerical simulations have been conducted for zirconium nitride (ZrN), the other main metal nitride proposed as a valuable material in nanooptics, with very similar conclusions as TiN. ZrN outperforms TiN for the near-field enhancement by a factor of 10 (see Figs 3b and 4b). However, the near-field enhancement of ZrN is still one order of magnitude lower than that of gold, especially at resonance where the relative efficiency is the worst (around 4% at resonance, see Fig. 4c). The photothermal properties of TiN and ZrN are very similar, as observed by comparing Figs 3e and 4e.

Maps (c) and (f) in Figs 3 and 4 are valuable to compare the near-field enhancement, and the photothermal efficiencies separately via the figures of merit Fa and Δ*T*. However, Fa and Δ*T* are not the only figures of merit than can be used in plasmonics. Depending on the application, other sensible figures of merits can be Fa, Jo^38^, Δ*T*, *σ*_abs_, *σ*_abs_/*V*, 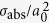, etc. This article is intended to more specifically discuss the main applications of metal nitrides, such as HAMR. In this kind of application, the optical near-field is the physical parameter of interest and a temperature increase is detrimental. Although the optical near-field of a metal nanoparticle is proportional to the incident light intensity, it cannot be as large as desired due to photothermal effects and possible thermal damage of the nanoparticle. In other words, Fa has to be high in order to achieve large near-field enhancement and Δ*T* has to be reduced in order to prevent thermal damage or artifactual thermal-induced effects, even under high light intensity. Thus, a relevant figure of merit in such applications is Fa/Δ*T*. But this quantity is not dimensionless. It is a near-field enhancement per unit Kelvin (K^−1^). Its absolute value is hard to interpret and we propose to use a refined dimensionless figure of merit defined by the relation.


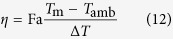


where *T*_amb_ is the ambient temperature and *T*_m_ is the melting temperature of the material. This way, the melting point of the material is taken into account in the figure of merit, which makes more sense. The higher *T*_m_, the better the figure of merit. Moreover, the interest of this figure of merit is that it is dimensionless and it has a physical meaning. It represents the maximum near-field enhancement that can be achieved before the structure melts. In other words, it is the near-field enhancement obtained when the temperature of the structures reaches the melting temperature (Δ*T* = *T*_m_ − *T*_amb_). Note that the presence of *T*_amb_ in the definition of *η* implies some arbitrariness in its definition. In this article we consider *T*_amb_ = 20 °C but any other pertinent choice would not change the results as *T*_m_ − *T*_amb_ ≫ *T*_amb_ (see values of *T*_m_ mentioned in the introduction of this article for Au, TiN and ZrN).

In the definition of *η*, we chose to consider the melting temperature, but what really matters in practice is rather the reshaping temperature where most of the experimental problems primarily occur. Thus, it would make more sense to normalize with the reshaping temperatures. However, while the reshaping temperature of Au nanoparticles has been widely investigated, the reshaping temperatures of metal nitride nanoparticles are not determined. Moreover a reshaping temperature is not something universal that only depends on a material. For gold different reshaping temperatures have been reported, from 100 °C and 400 °C. Thus, we prefer to deal with the melting temperatures, which makes sense as well if one assumes that the reshaping temperature of nanoparticles varies in proportion with the melting temperature.

**Figure 5:**
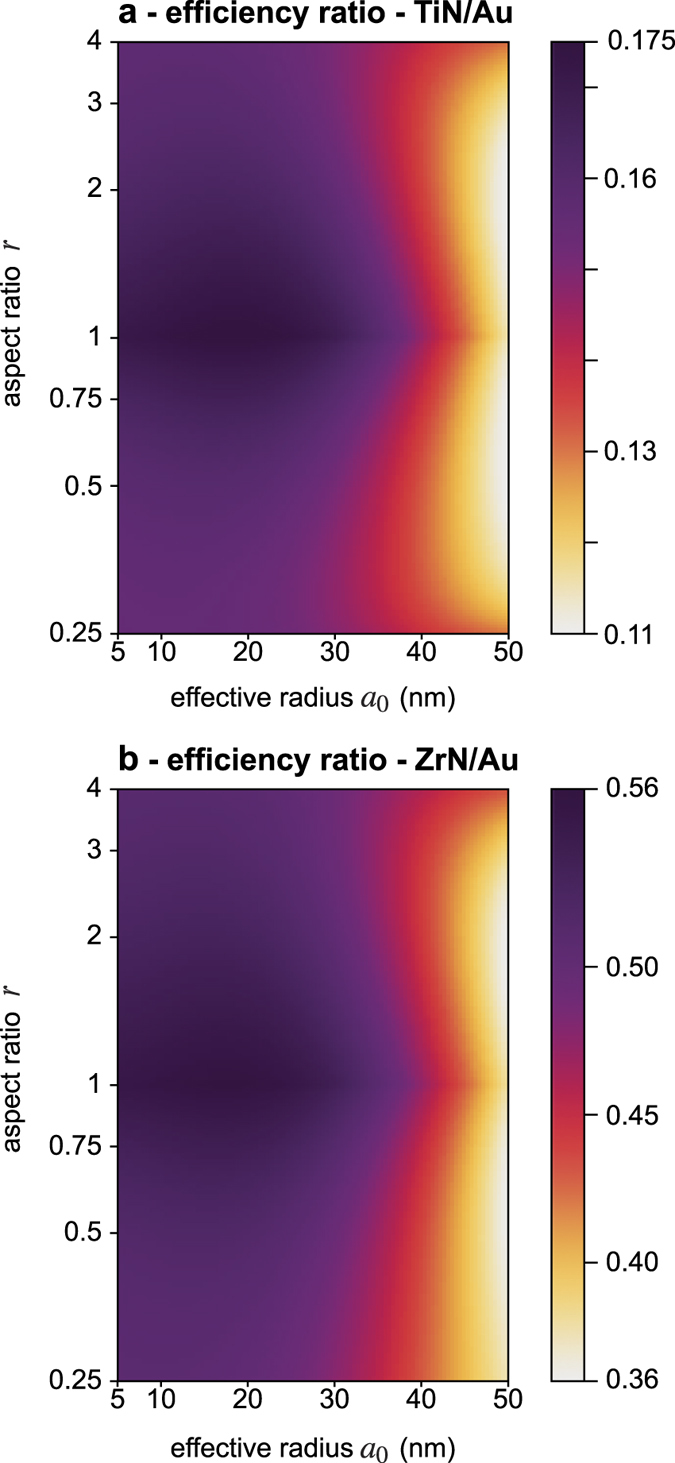
Maps of the efficiencies *η* of TiN and ZrN normalized by the efficiency *η* of Au, defined.

Figure 5 represents the maps of *η*_TiN_/*η*_Au_ and *η*_ZrN_/*η*_Au_ at *λ* = 800 nm, where *η*_X_ is the *η* value for the material X. These maps consist of pixel-by-pixel ratios between maps (c) and (f) in Figs 3 and 4, multiplied by the quantity *T*_m_ − *T*_amb_. They provide a quantitative estimation of the efficiencies of TiN and ZrN with respect to gold in HAMR. According to these results, in the infrared, the efficiency of TiN does not exceed 18% of the efficiency of gold (Fig. 5a). The signification of this value is important. It means that near the melting points of each material (2930 °C for TiN and 1064 °C for gold), the near-field enhancement of a TiN structure does not exceed 18% of the near field enhancement achieved by a gold structure. Regarding ZrN, this efficiency is higher but does not exceed 60% (Fig. 5b). Thus, over the space of parameters investigated in our work, ZrN seems to be a better nanoplasmonic material than TiN but does not outperform gold.

Another conclusion can be drawn from these maps: enlarging or elongating metal nitride particles decreases the efficiency of these material compared to gold. The best efficiencies are obtained with small spherical TiN and ZrN particles. This conclusion is also detrimental for applications such as HAMR, where large and sharp structures are usually considered.

All the simulations presented in this article have been conducted at *λ* = 800 nm, as it is the most common wavelength used in applications such as HAMR^28^,^29^,^30^,^31^,^32^,^33^. We also conducted numerical simulations at 500 nm and 650 nm (see Supplementary Figs S1–S4). Results at 650 nm yield similar conclusions compared to 800 nm. At 500 nm though, ZrN is shown to outperform gold by a factor of 4 (see Supplementary Fig. S5). But this is mainly because gold becomes a poor plasmonic material in the blue-UV due to a strong increase of the imaginary part of the permittivity *ε*″ (see Fig. 2b) and the occurrence of interband transitions that favor losses from around *λ* = 500 nm. ZrN becomes artificially better for this reason, but in the UV-blue range, the efficiencies *η* of both gold and metal nitrides decrease dramatically, as further illustrated by Supplementary Fig. S6.

## Discussion

We shall now discuss the limits of our model, as some simplifications have been made compared to a real operating system.

In practical HAMR applications, the near-field interaction of the particle with the surface of the magnetic disc, usually metallic, should be taken into account. This interaction can be described by using *e.g.* the image dipole formalism. This coupling depends on the nature of the surface and the gap size, which are specific to each application. The study of this effect falls beyond the scope of the present work, but we believe that the strong tendencies derived herein should not be overturned.

All these results question the interest of metal nitrides for applications in plasmonics where a temperature increase is detrimental and likely to happen, despite their high melting temperature. Although the range of morphologies is limited in our work (spheroids), we believe these conclusions to be solid since we address both essential aspects of the design of plasmonic structures: size and asymmetry. The only morphological aspect that we do not address is the presence of a plasmonic gap.

Our approach also relies on optical constant data sets from Johnson & Christy^41^ and from Palik’s Handbook^42^. Guler and coworkers have recently shown that the optical constants of TiN may vary depending on the sample temperature during deposition, because of a modification of the nitrogen/metal ratio and presumably of the surface roughness^39^. Although this modification is not dramatic, further investigations could address the problem of the nanoplasmonic efficiency of TiN and ZrN as a function of the metal/nitride ratio.

In addition, the mechanism of energy transfer in HAMR is not clearly identified. A near-field radiative transfer is also possible, although it has only been occasionally mentioned in the community. This effect concerns different physical phenomena that are not taken into account in our model.

Although this work gives pessimistic conclusions regarding the interest of metal nitrides for some applications in nano-optics, this work does not definitely rule on whether metal nitrides are of interest or not in applications such as HAMR. Answering such a question would require extensive numerical simulations on a large variety of geometries of the system (writing head, magnetic disk), the presence of a magnetic field, the effect plasmon-phonon interaction, etc. It would even be difficult to fully address this problem and definitely answer the question in a single article. This article is rather intended to help researchers and companies that are working on metal nitride nanoparticles in nano-optics to build a good physical picture and intuition, to guide them in their studies and to help them understand and interpret the results of their experimental or numerical investigations. Also for this reason, all the Matlab codes used in this work are provided in Supplementary Information. For instance, they could be used to plot results as presented in Figs 3 and 4 to compare other materials with gold, or investigate other geometries.

In summary, we have investigated the properties of two metal nitrides, TiN and ZrN, for applications in nano-optics. The aim was to give a solid physical picture to help answer the question “to what extent can metal nitrides outperform gold in nanooptics?”. Because playing with the resonance of nanoparticles essentially consists in playing with shape and size, we focused on spheroid nanoparticles of various sizes and aspect ratios. This way, we could address the effects of retardation and asymmetries. We focused on two physical quantities, which are the quantities of interest in most applications of nano-optics and plasmonics: the near-field enhancement and the temperature increase of the nanoparticles. This work was also the occasion to explain how the temperature increase of spheroid plasmonic nanoparticles can be easily determined using close-form expressions, and to define a new figure of merit in plasmonics, named *η* in this article. It emerged that metal nitrides are poor near-field enhancers compared to gold. The best efficiencies in term of near-field enhancement of metal nitrides compared to gold are obtained for small spherical nanoparticles and still do not exceed 60%. Deviating from spherical metal nanoparticles makes metal nitrides even less efficient. In this respect, metal nitrides do not stand for good candidates for applications where the optical near-field matters and where a temperature increase is detrimental, such as heat assisted magnetic recording (their main envisioned application), surface enhanced Raman scattering or plasmon-assisted photochemistry. Regarding the thermoplasmonic properties of metal nitrides, the results showed that they are efficient heat sources under illumination, with equivalent abilities compared to gold. They even outperform gold for most geometries, except at the gold nanoparticle plasmonic resonance. For this reason metal nitride stand for promising candidates for applications in thermoplasmonics, where heating is desired, *e.g.* photothermal therapy, photothermal imaging, photoacoustic imaging, thermophotovoltaics. Their wide wavelength range of absorption makes them particularly valuable for applications based on solar absorption.

## Methods

Numerical simulations of absorption cross sections and near-field enhancements were conducted using the MNPBEM Matlab toolbox developed by Hohenester and Trügler in 2012^40^. This package allows one to simply conduct numerical simulations in plasmonics using the Boundary Element Method, previously introduced in this field of research by García de Abajo in 2002^47^. The spheroid mesh was created using Blender^48^ and consisted of 1280 triangular faces. All the Matlab codes used in this work, including the MNPBEM main files, are provided in Supplementary Materials.

## Additional Information

**How to cite this article**: Lalisse, A. *et al*. Plasmonic efficiencies of nanoparticles made of metal nitrides (TiN, ZrN) compared with gold. *Sci. Rep.* **6**, 38647; doi: 10.1038/srep38647 (2016).

**Publisher's note:** Springer Nature remains neutral with regard to jurisdictional claims in published maps and institutional affiliations.

## Supplementary Material

Supplementary Information

Supplementary Dataset 3

Supplementary Dataset 1

Supplementary Dataset 2

Supplementary Dataset 4

Supplementary Dataset 5
