# Supplementary material for: Plasmonic efficiencies of nanoparticles made of metal nitrides (TiN, ZrN) compared with gold
Source: Sci Rep. 2016 Dec 9;6:38647. doi: 10.1038/srep38647 (PMC5146670; doi:10.1038/srep38647)
Supplement: Supplementary Information [file srep38647-s1.pdf]

Plasmonic efficiencies of nanoparticles made of metal nitrides (TiN, ZrN) compared with gold

Adrien Lalisse,[1,2] Gilles Tessier,[2] Jérome Plain,[1] Guillaume Baffou,[3,*]

[1]LNIO/ICD, UMR 6281, CNRS, Technological University of Troyes, 10004 Troyes, France
[2]Laboratoire de Neurophotonique UMR8250, CNRS, Faculté des sciences biomédicales et fondamentales, Université Paris Descartes, 75270 Paris, France
[3]Institut Fresnel, CNRS, Aix Marseille Univ, Centrale Marseille, Marseille, France

*guillaume.baffou@fresnel.fr

SUPPLEMENTARY INFORMATION

1 Matlab Codes Using the MNPBEM package

- `spheroid_NF.xml`.
  Matlab program that computes the maximal near-field achieved over a spheroid nanoparticle nanoparticle surface, and the associated Faraday number Fa. The extension `.xml` has to be replaced with `.m` for proper use.

- `spheroid_DT.xml`.
  Matlab program that computes the temperature increase ΔT of a spheroid nanoparticle.

- `TiN.xml`, `ZrN.xml` and `Au.xml`.
  Optical constants of TiN, ZrN and Au. The three columns are energy (in eV), real part of the refractive index and imaginary part of the refractive index.The extension `.xml` has to be replaced with `.txt` for proper use.

- `sphere4.xml`
  Spherical mesh of radius 1 generated using Blender and composed of 1280 triangles.The extension `.xml` has to be replaced with `.txt` for proper use.

2 Figures

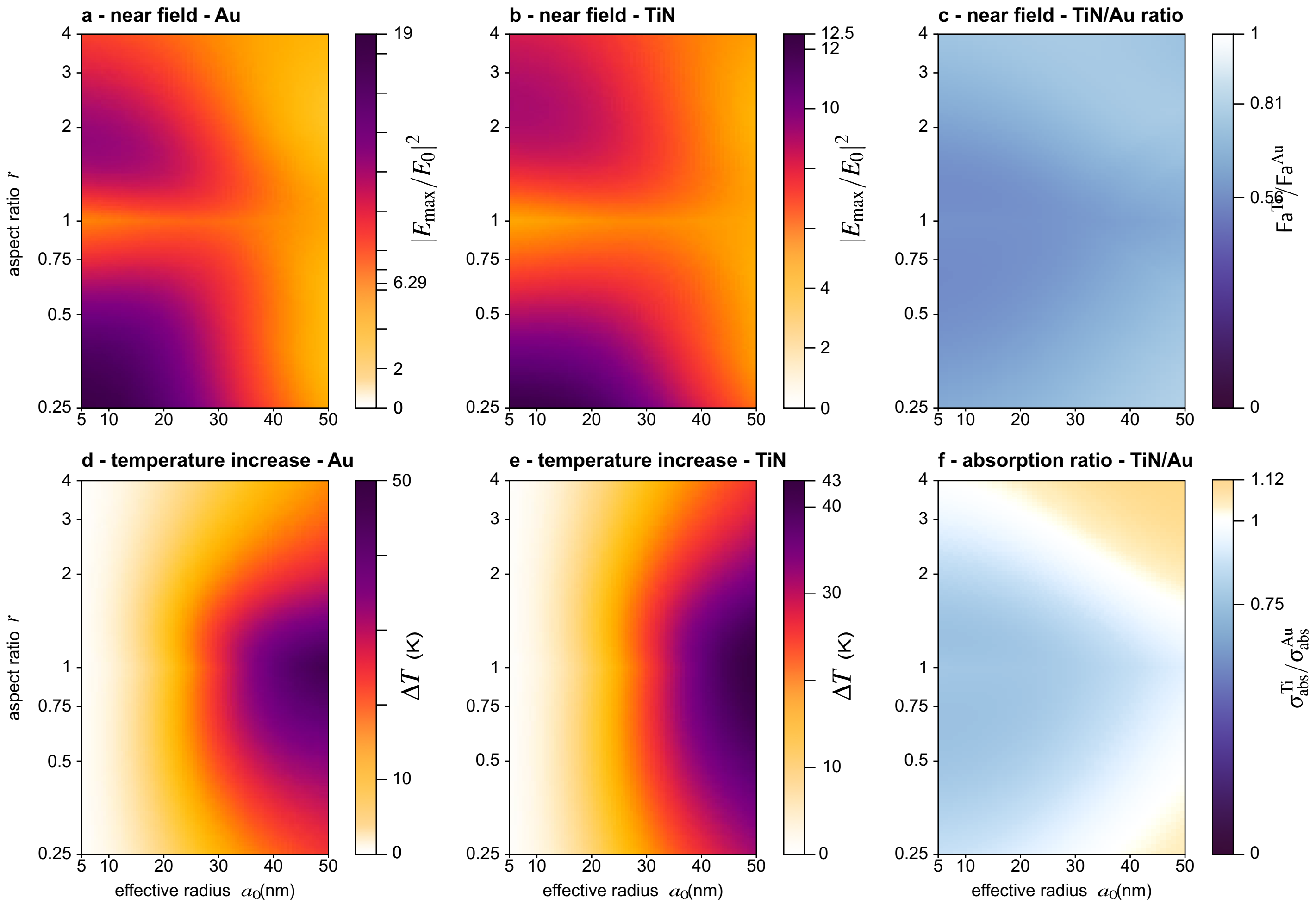

Fig. S1 Properties of Au and TiN spheroids as a function of the aspect ratio r and the equivalent radius a_0, under illumination at $\lambda = 500$ nm. (a) Near-field enhancement of Au spheroids. (b) Near-field enhancement of TiN spheroids. (c) Ratio of the data represented in images (b) and (a) respectively. (d) Temperature increase of Au spheroids. (e) Temperature increase of TiN spheroids. (f) Ratio of the data represented in images (e) and (d), which amounts to representing the ratio of the absorption cross sections of TiN and Au spheroids.

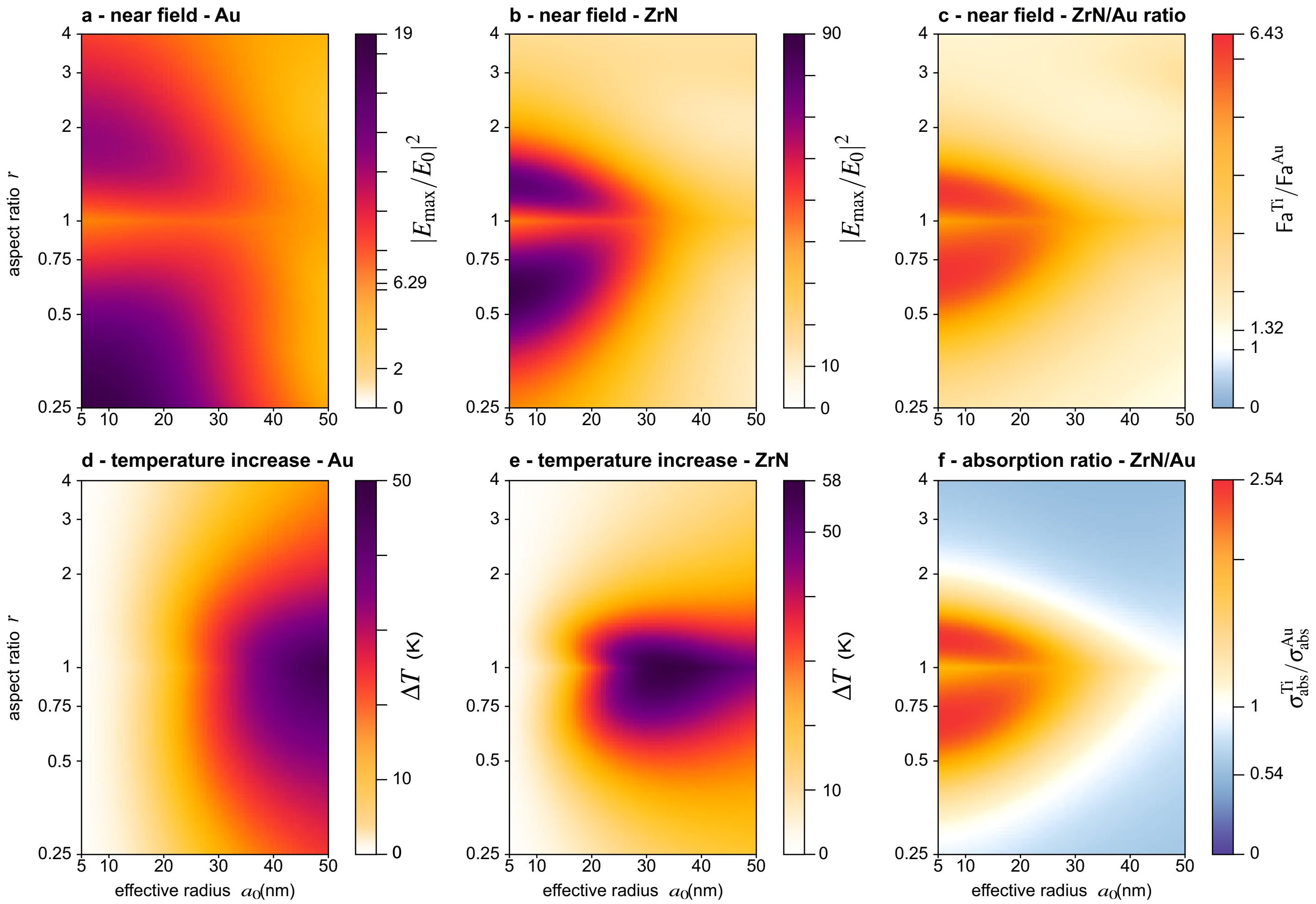

Fig. S2 Properties of Au and ZrN spheroids as a function of the aspect ratio r and the equivalent radius a_0, under illumination at $\lambda = 500$ nm. (a) Near-field enhancement of Au spheroids. (b) Near-field enhancement of TiN spheroids. (c) Ratio of the data represented in images (b) and (a). (d) Temperature increase of Au spheroids. (e) Temperature increase of ZrN spheroids. (f) Ratio of the data represented in images (e) and (d), which amounts to representing the ratio of the absorption cross sections of ZrN and Au spheroids.

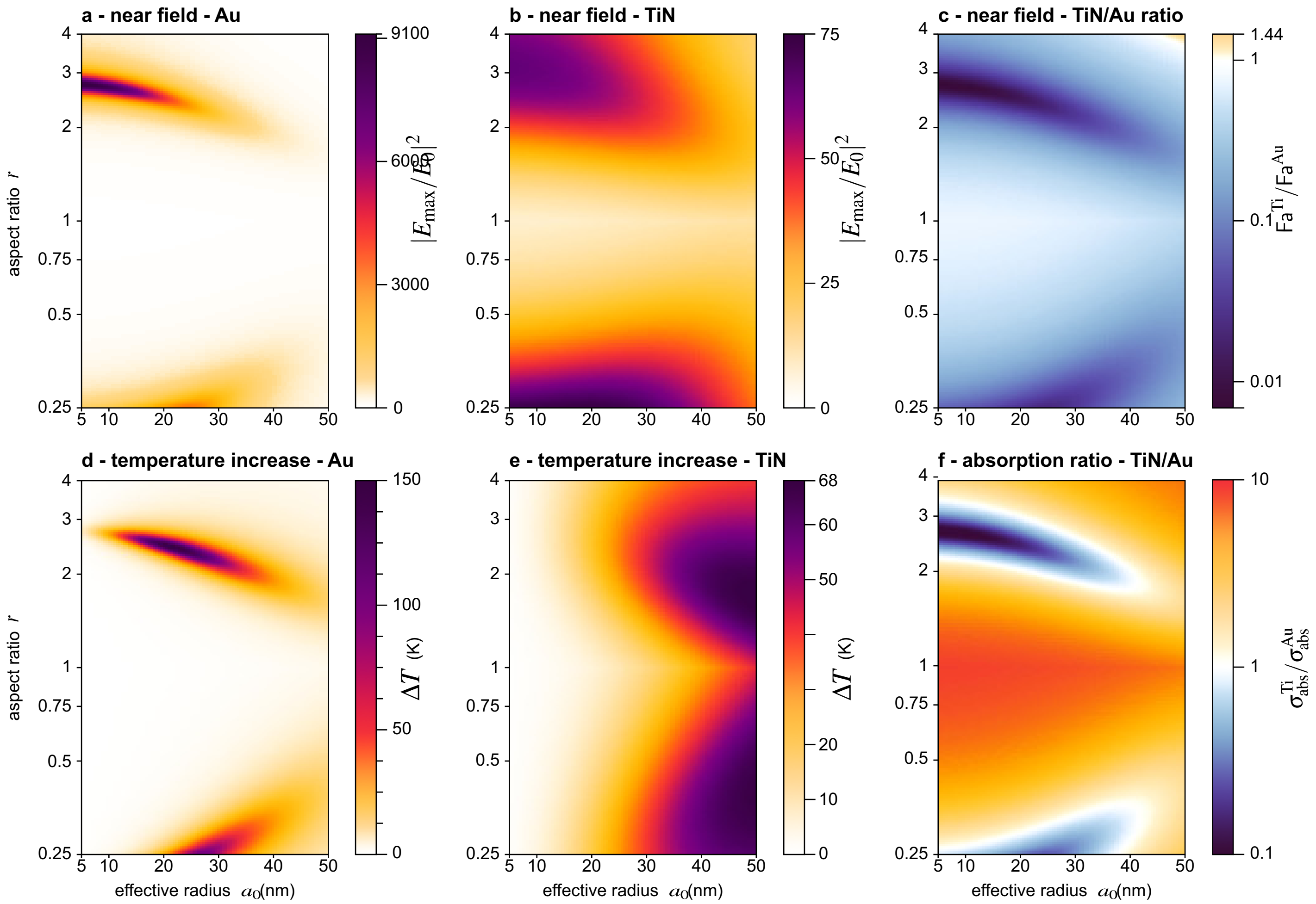

Fig. S3 Properties of Au and TiN spheroids as a function of the aspect ratio r and the equivalent radius a_0, under illumination at $\lambda = 650$ nm. (a) Near-field enhancement of Au spheroids. (b) Near-field enhancement of TiN spheroids. (c) Ratio of the data represented in images (b) and (a) respectively. (d) Temperature increase of Au spheroids. (e) Temperature increase of TiN spheroids. (f) Ratio of the data represented in images (e) and (d), which amounts to representing the ratio of the absorption cross sections of TiN and Au spheroids.

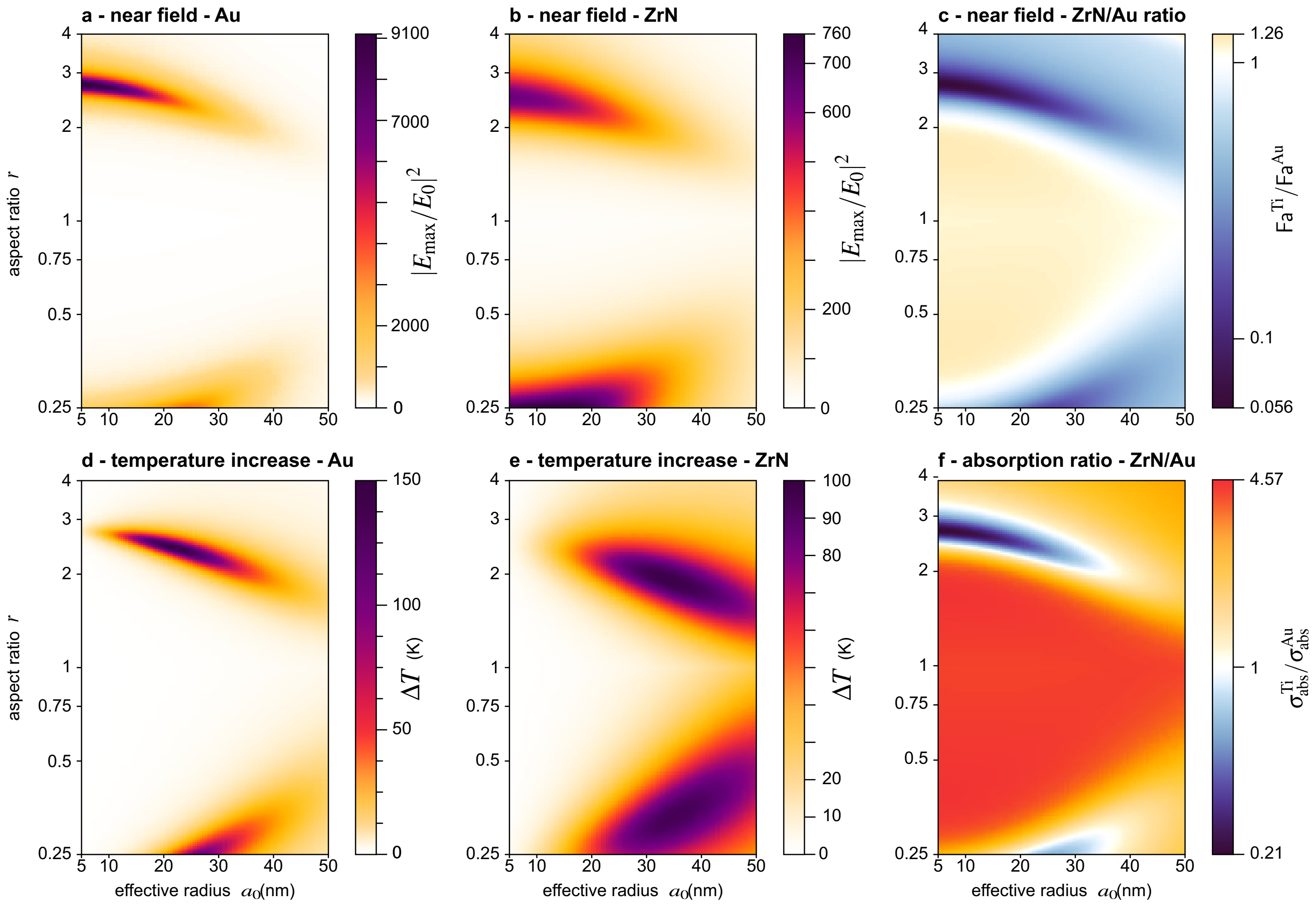

Fig. S4 Properties of Au and ZrN spheroids as a function of the aspect ratio r and the equivalent radius a_0, under illumination at $\lambda = 650$ nm. (a) Near-field enhancement of Au spheroids. (b) Near-field enhancement of TiN spheroids. (c) Ratio of the data represented in images (b) and (a). (d) Temperature increase of Au spheroids. (e) Temperature increase of ZrN spheroids. (f) Ratio of the data represented in images (e) and (d), which amounts to representing the ratio of the absorption cross sections of ZrN and Au spheroids.

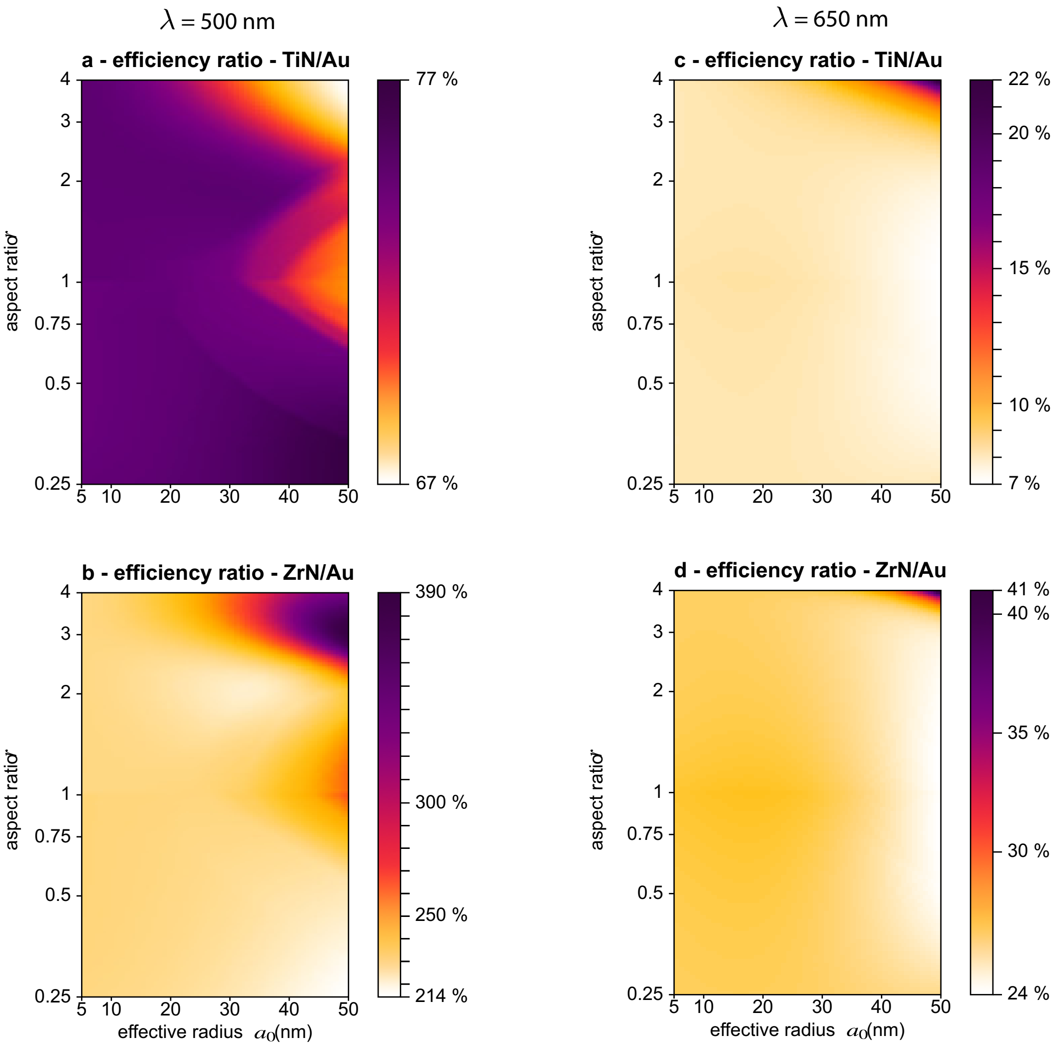

Fig. S5 Maps of the efficiencies η of TiN and ZrN normalized by the efficiency η of Au, as represented in Figure 4 of the manuscript for $\lambda = 800$ nm. (a) Case $\lambda = 500$ nm. (b) Case $\lambda = 650$ nm.

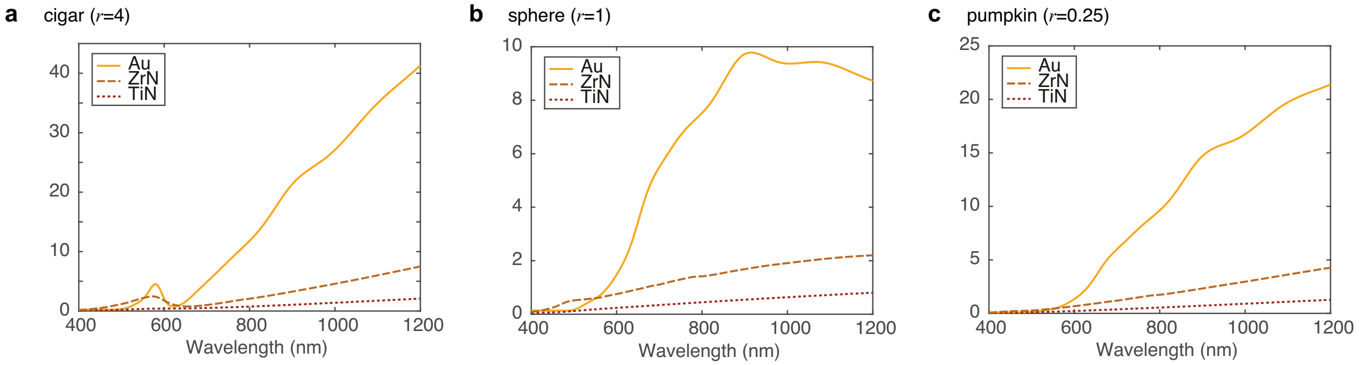

Fig. S6 Efficiencies η for gold and metal nitrides as a function of the wavelength for nanoparticles with an effective radius $a_0 = 50$ nm. (a) Nanoparticle aspect ratio $r = 0.25$ (pumpkin). (b) Nanoparticle aspect ratio $r = 1$ (sphere). (c) Nanoparticle aspect ratio $r = 4$ (cigare).
